# Rough and Tough: How Particle Surface Roughness Affects Liquid Marble Formation and Stability

**DOI:** 10.1002/advs.202501378

**Published:** 2025-04-07

**Authors:** Umair Sultan, Celin Kotulla, Kall Kefle, Syuji Fujii, Nicolas Vogel

**Affiliations:** ^1^ Institute of Particle Technology Department of Chemical and Biological Engineering Friedrich‐Alexander‐Universität Erlangen‐Nürnberg Cauerstrasse 4 91058 Erlangen Germany; ^2^ Department of Applied Chemistry Faculty of Engineering Osaka Institute of Technology 5‐16‐1, Omiya, Asahi‐ku Osaka 535–8585 Japan

**Keywords:** liquid marbles, mechanical testing, stability, supraparticles, surface roughness, wetting

## Abstract

Liquid marbles are liquid droplets encased by non‐wetting particles. They exhibit elastic and non‐sticking properties that enable applications such as sensors, adhesives, miniature reactors, and material carriers. The formation, stability, and properties of liquid marbles depend on the physico‐chemical characteristics of the solid particles. This study systematically explores the impact of particle surface roughness on liquid marbles by employing colloidal supraparticles as well‐defined model systems. Supraparticles are spherical aggregates of uniform colloidal primary particles, which enable adjusting the characteristic surface roughness by varying the primary particle size. Increasing surface roughness increases the interfacial contact angle, which, in turn, influences the mechanical properties and liquid marble stability. The presence of surface roughness increases the deformation resistance of the liquid marble, which counteracts the spreading of the inner liquid upon mechanical impact, and therefore hinders rupture. The increased contact angle further enables the formation of liquid marbles from increasingly low‐surface‐tension organic liquids. This study thus provides detailed insights into the structure‐property relationships governing the preparation of stable liquid marbles based on particle surface characteristics.

## Graphical abstract

This study investigates the influence of particle surface roughness on the formation and stability of liquid marbles using well‐defined colloidal supraparticles as model systems. Increasing surface roughness enhances the interfacial contact angle, improves mechanical stability by increasing deformation resistance upon impact, and enables liquid marble formation from low‐surface‐tension organic liquids.

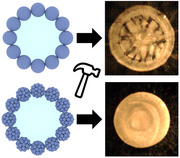

## 1 Introduction

Liquid marbles (LMs) are liquid droplets coated with solid, non‐wetting particles that adsorb onto the air‐liquid interface, stabilizing the droplet and preventing it from wetting external surfaces.^[^ ^1^, ^2^, ^3^, ^4^, ^5^, ^6^ ^]^ This coating thus imparts elastic and non‐sticking properties to a liquid, allowing facilitated handling and transport. Upon exposure to external stimuli,^[^ ^7^ ^]^ such as pressure,^[^ ^8^ ^]^ heat,^[^ ^9^ ^]^ light,^[^ ^10^ ^]^ or chemical changes,^[^ ^11^ ^]^ or by pre‐programming,^[^ ^12^ ^]^ LMs can be triggered to rupture on demand, releasing their internal liquid. These properties make them attractive for applications such as sensors,^[^ ^13^, ^14^, ^15^ ^]^ microactuators,^[^ ^16^, ^17^ ^]^ pressure‐sensitive adhesives,^[^ ^18^ ^]^ material carriers,^[^ ^19^ ^]^ microfluidics,^[^ ^20^, ^21^ ^]^ and miniature reactors.^[^ ^22^, ^23^ ^]^

The formation of LMs and their resultant mechanical properties are determined by the physico‐chemical properties of the stabilizing particles.^[^ ^3^, ^24^ ^]^ To form aqueous LMs, the stabilizing particles need to be hydrophobic, so that they form a solid layer protecting the underlying liquid upon interfacial adsorption.^[^ ^1^, ^25^, ^26^ ^]^ Forming LMs from non‐polar organic liquid is more challenging due to their lower surface tension, which inherently lowers the interfacial contact angle of the stabilizing particles.^[^ ^27^, ^28^ ^]^

The size and morphology of the stabilizing particles determine their arrangement on the surface of an LM, and thus their mechanical properties.^[^ ^29^, ^30^, ^31^, ^32^ ^]^ Smaller particles (≤5 µm) tend to form ill‐defined multilayer aggregates on the droplet surface, which enhances their impact resistance compared to larger, smooth particles.^[^ ^32^ ^]^ Larger particles form monolayers with increasing order that stabilize the LM.^[^ ^30^ ^]^ In such monolayer‐based LMs, the mechanical stability increases with particle size due to stronger capillary forces between the particles and a larger gap between the inner liquid and the external surface.^[^ ^29^, ^30^ ^]^ The particle shape also affects the mechanical behavior. Spherical particles allow for elastic behavior, meaning the LM can recover its shape after deformation, while anisotropic, rod‐shaped particles lead to a more plastic response due to an increase in the packing degree under stress.^[^ ^31^ ^]^ In an extreme case, platelet‐shaped particles enable the design of anisotropic LMs by efficiently jamming the liquid interface.^[^ ^33^ ^]^

The stabilization of LMs formed by liquids with lower surface tension requires engineering the particle surface. To increase the contact angle and thus prevent wetting, fluorinated surface chemistries, and particle surface structure need to collude to provide sufficiently high contact angles.^[^ ^28^, ^34^, ^35^, ^36^ ^]^ This can be generally achieved by exploiting the tendency of small particles (<≈100 nm) to adsorb to the liquid interface in the form of aggregates, which induce an ill‐defined nanoscale surface roughness.^[^ ^28^, ^35^ ^]^ Hierarchical particle systems, such as polymer plates structured with microparticles (≈2 µm) provide a similar effect and enable the formation of polyhedral LMs using low‐surface‐tension alkanes.^[^ ^36^ ^]^

While these studies underscore the critical role of surface roughness on the formation and stability of LMs, the ill‐defined nature of the surface roughness of available powder particles prevents the establishment of systematic structure‐property relations.

Here, we use colloidal supraparticles (SPs) as model systems to investigate the effect of particle surface roughness on LM formation and stability in detail. SPs are defined spherical aggregates of smaller, colloidal building blocks called primary particles.^[^ ^37^ ^]^ They are fabricated by the confined assembly of these primary particles within droplets, which can be achieved by spray‐drying,^[^ ^38^, ^39^ ^]^ emulsion templating,^[^ ^40^, ^41^ ^]^ or by drying on liquid repellent surfaces.^[^ ^42^, ^43^, ^44^ ^]^ SPs exhibit additional functionalities inherited by synergistic combinations of primary particles,^[^ ^45^ ^]^ or via emergent properties resulting from the ordered arrangement of primary particles.^[^ ^37^ ^]^ The latter gives rise to structural color from interference effects,^[^ ^46^, ^47^ ^]^ tunable, hierarchical porosity^[^ ^48^, ^49^, ^50^, ^51^ ^]^ or controlled surface roughness.^[^ ^52^ ^]^

The surface roughness is directly determined by the size of the primary particles, which form a defined topography at the surface of the SP. Changing the size of primary particles forming the SP thus allows systematically varying the surface roughness. In this study, the radius of the primary particles defines the surface roughness features protruding from SP surface. By varying the diameter of the silica primary particles from 240  to 970 nm, we systematically control the characteristic dimensions of the surface roughness features in SP‐based powders. Using these model particles, we explore how surface roughness affects the contact angle, the ability to stabilize LMs from low‐surface‐tension liquids, and the mechanical stability upon compression and impact.

## 2 Results and Discussion

**Figure 1:**
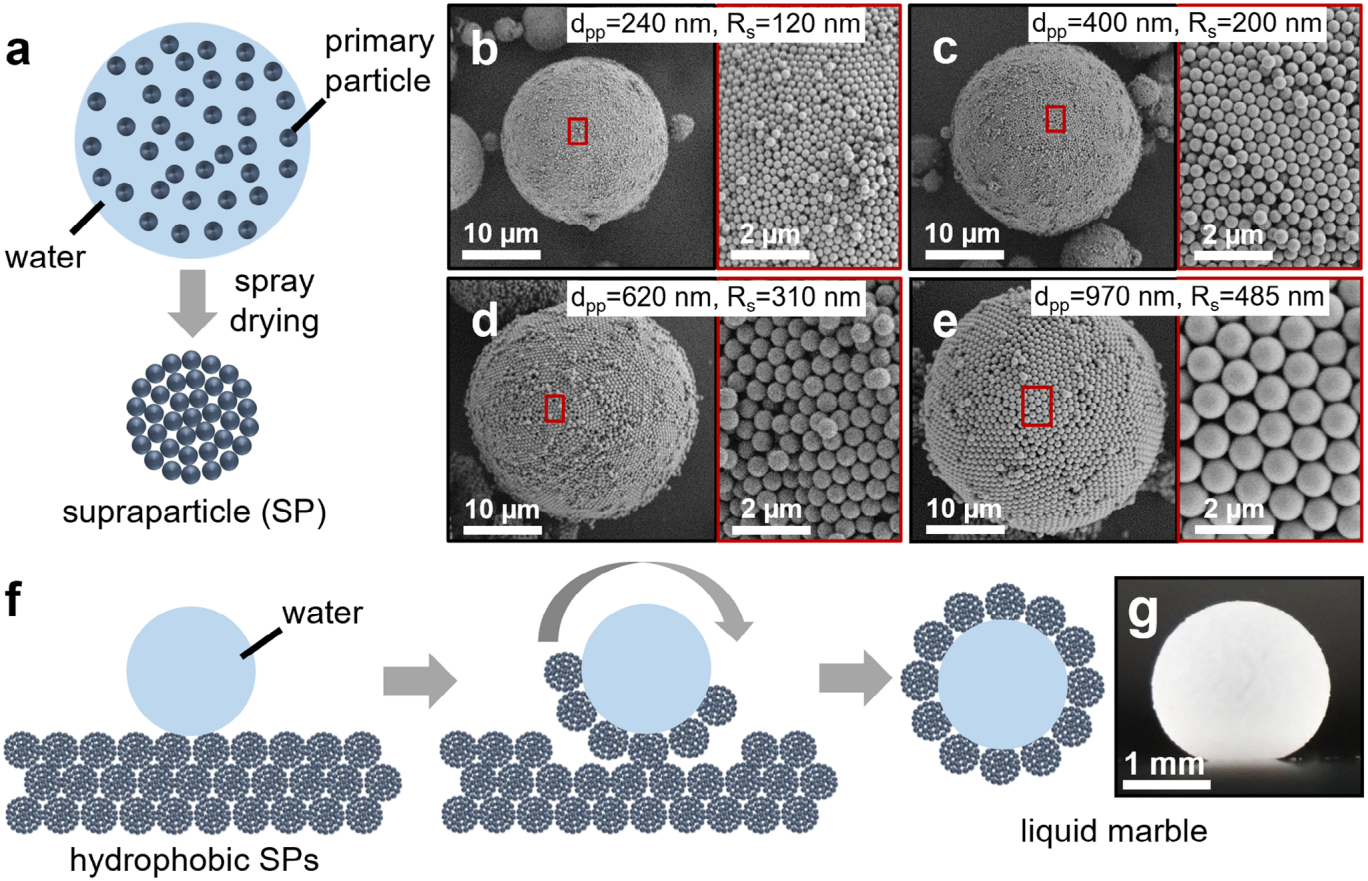
Fabrication of supraparticles and liquid marbles. a) Schematic illustration of the formation of supraparticles by spray drying. b–e) SEM images of supraparticles with primary particle diameter (d_pp_) and surface roughness (R_s_) of: (a) d_pp_ = 240 nm, R_s_ = 120 nm; (b) d_pp_ = 400 nm, R_s_ = 200 nm, (c) d_pp_ = 620 nm, R_s_ = 310 nm; and (d) d_pp_ = 970 nm, R_s_ = 485 nm. Insets show the increase in surface roughness with increasing primary particle diameter. f) Schematic illustration of the liquid marble formation by rolling a liquid droplet on powder bed. g) Exemplary photograph of a formed aqueous liquid marble, using a supraparticle powder with R_s_ = 485 nm.

We start by fabricating SPs with tailored surface roughness. To achieve this, we use the Stöber process^[^ ^53^ ^]^ to synthesize colloidal silica primary particles with average diameters ranging from 240 to 970 nm, while maintaining narrow size distributions (Figure S1, Supporting Information). These primary particles are then consolidated into SPs through spray‐drying (**Figure** **1**a), producing well‐defined, spherical SPs (Figure 1b–e).^[^ ^54^, ^55^ ^]^ We ensure that all the samples have comparable size distributions and average diameters of ≈13 µm to minimize potential size‐dependent effects on LM formation (Figure S2, Supporting Information). The consolidated SPs exhibit a surface composed of close‐packed primary particles, so the characteristic dimension of surface roughness scales directly with the diameter of the primary particles,^[^ ^52^ ^],^ i.e., larger primary particles yield SPs with higher surface roughness. We define this characteristic surface roughness as the height of the primary particles protruding from the surface of the supraparticle. By assuming that the actual supraparticle surface is the region where the outmost layer of primary particles are in close contact, the roughness thus corresponds to the radius of the primary particles (Figure S2f, Supporting Information). Thus, the surface roughness of supraparticles formed from primary particles with diameters of 240, 400, 620, and 970 nm is defined as 120, 200, 310, and 485 nm, respectively. This surface roughness is evident in representative SEM images, where the ordered surface structures consisting of close‐packed primary particles protruding from the SP surface are readily observed (Figure 1b–e). Note that the individual primary particles that adsorb onto the supraparticle surface during the spray‐drying process contribute additional roughness beyond the nominal roughness defined by the primary particle size. These satellite particles are present in all powder samples. For simplicity, we therefore use the nominal roughness provided by the primary particles to characterize the different powders.

As a reference particle system without notable surface roughness, we use a mix of commercial smooth silica microparticles with diameters of 2, 6, 11, 20 µm that mimics the size distribution of the SP samples (Figure S2, Supporting Information). The reference particles exhibit a smooth surface in the SEM analysis (Figure S2a, Supporting Information), and we therefore assign them a roughness value of 0 nm. We acknowledge that this is an oversimplification, since the actual sample may have (sub)nanometer‐scale surface fluctuations, but it provides a clear contrast to SP powders, where roughness is defined by the particle radius. All powders are surface‐modified using heptadecafluorodecyl‐trichlorosilane to increase the contact angle with both aqueous and hydrocarbon liquids. We then form aqueous LMs from all powder samples by rolling a droplet of specific volume (5 µL) on a powder bed for 40 s (Figure 1f,g). During the rolling process the particles adsorb on to air‐water interface and coat the droplet completely. To remove excess particles all LMs are rolled on a bare glass surface for 30 s before use.

**Figure 2:**
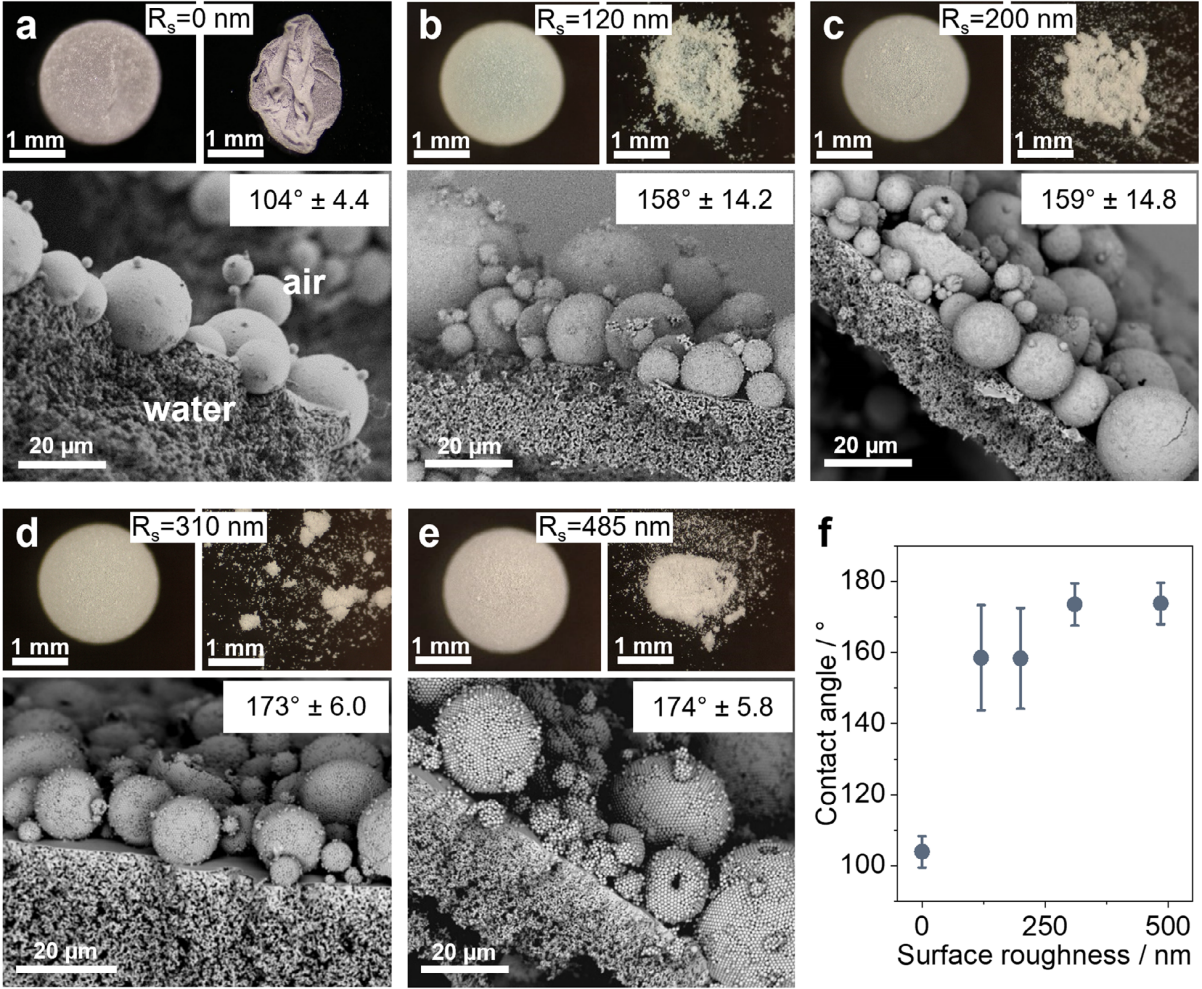
Stereomicroscopy and contact angle analysis of aqueous liquid marbles. a–e) Top: stereomicroscopy images, before (left) and after (right) drying, and bottom: SEM analysis of the air‐water interface of liquid marbles formed using smooth particles (R_s_ = 0 nm) and supraparticles of surface roughness (R_s_) 120, 200, 310, and 485 nm, respectively. f) Contact angle of particles at the air‐water interface as a function of surface roughness. The contact angle value is an average of at least 5 different particle measurements for every sample. The error bar represents the standard deviation of these measurements.

We analyze the composition of LMs by measuring the mass of particles after water evaporation. For all samples, the determined particle loading on the surface of the LM was below 7 wt.%. We use this solid fraction to estimate the numbers of particle layers on the surface of the LMs. This yields a value between 1.1 and 1.4, indicating that the LMs are primarily covered with a monolayer of particles, and not with ill‐defined multilayers (Table S1, Supporting Information), a prerequisite to isolate the effect of surface roughness. Stereomicroscope analysis of dried LMs shows that for the case of smooth particles, the particle shell retains a buckled structure after drying (**Figure** **2**a, top). The opposite is true for rough‐particle shells, which disintegrate after drying (Figure 2b–e, top). Since the chemical composition of particles and particle surfaces is identical, and both types of samples exhibit a similar surface coverage, we propose that this difference in drying behavior must be caused by differences in their interfacial position.

To visualize the particle position at the LM surface, we fix the air‐water interface by exposing the LMs to ethyl‐2‐cyanoacrylate (Super‐Glue) vapor (Figure S3, Supporting Information).^[^ ^56^ ^]^ This monomer undergoes anionic polymerization upon contact with water to form a polymer membrane around the particles and thus fixes their positions. After removing residual water by evaporation, the solidified air‐water interface is analyzed in SEM (Figure 2a–e, bottom). This method confirms that the LM surfaces are generally covered by a monolayer of absorbed particles with only a few extra particles sticking to these interfacially‐adsorbed SPs. We geometrically determine the water contact angle of the particles from the SEM images by manually defining the particle boundary and the interface in an image processing software as shown in Figure S4 (Supporting Information). We observe an increase in water contact angle with increasing particle surface roughness (Figure 2f), which is consistent with the Cassie‐Baxter wetting model.^[^ ^57^ ^]^ The hydrophobic surface chemistry induced by the fluorosilanization, combined with increased surface roughness, results in superhydrophobic particles with contact angles exceeding 150°.

We can now correlate the observed difference in the cohesion behavior of dried LMs with the difference in particle contact angles. As the desorption energy of the particles is a function of the contact angle,^[^ ^58^, ^59^ ^]^ rough particles, which only touch the water surface will exhibit a lower desorption energy and can therefore be expelled from the shrinking water surface, leaving a disintegrated powder after drying. In contrast, smooth particles, with lower contact angles, are embedded deeper into the water, resulting in higher desorption energies.^[^ ^60^ ^]^ Moreover, the comparatively larger contact line radius leads to stronger lateral capillary forces, since the length of the liquid meniscus formed at the contact points is smaller.^[^ ^61^, ^62^ ^]^ This translates into higher cohesion between smooth particles, which prevents the desorption of excess particles as the interface shrinks. Instead, the particle surface layer buckles upon drying without disintegration.

**Figure 3:**
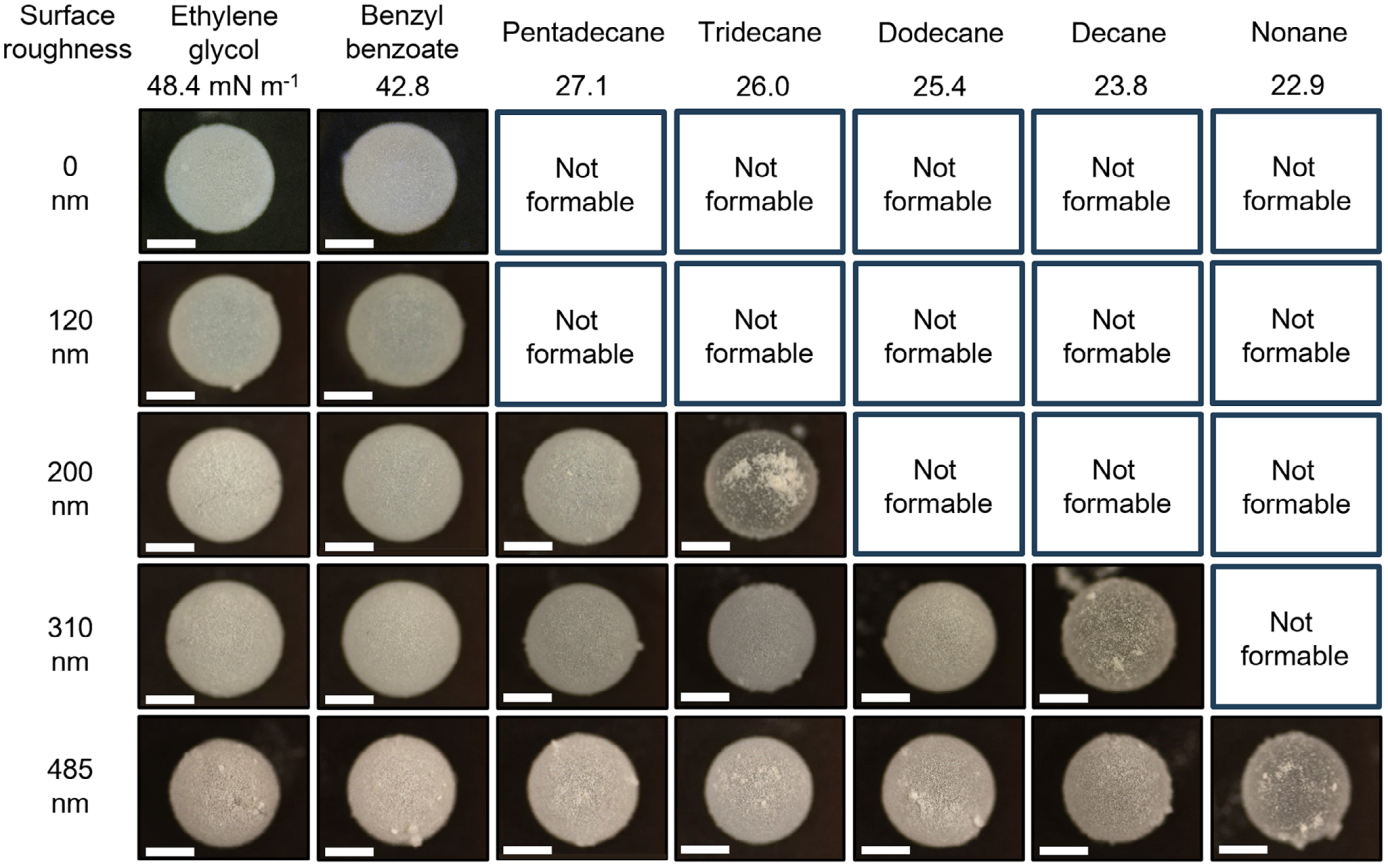
Formation of organic liquid marbles from particles with varying surface roughness and liquids with different surface tensions. With increasing surface roughness, liquids with lower surface tensions can be stabilized. All scale bars are 1 mm.

We now evaluate the ability to form LMs with low‐surface‐tension organic liquids as a function of surface roughness. We use liquids with decreasing surface tension γ, starting from ethylene glycol (γ  =  48.4 mNm^−1^) to nonane (γ  =  22.9 mNm^−1^) and map which SP powders can stabilize the LM. **Figure** **3** shows that each particle system exhibits a threshold in surface tension below which stable LMs cannot be formed. Smooth particles form LMs with ethylene glycol (γ  =  48.4 mNm^−1^) and benzyl benzoate (γ  =  42.8 mNm^−1^), but fail with lower surface tension liquids, establishing a threshold of ≈40 mN m^−1^. Similar results are obtained for SPs with surface roughness of 120 nm. SPs with a larger surface roughness of 200  and 310 nm are able to stabilize LMs with tridecane (γ  =  26.0 mNm^−1^) and decane (γ  =  23.8 mNm^−1^), respectively. The SP powder with the largest surface roughness features of 485 nm enabled the formation of LMs with nonane (γ  =  22.9 mNm^−1^). These results demonstrate that there is a direct correlation between the magnitude of particle surface roughness and surface tension of the liquids that can be stabilized in the form of an LM.

**Figure 4:**
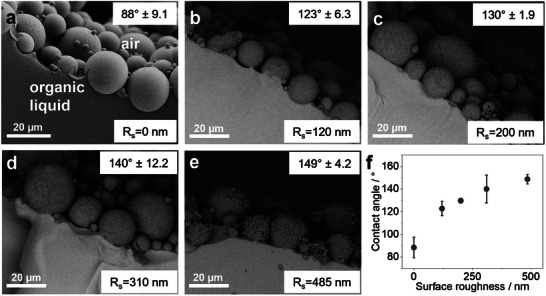
Visualization of the air‐liquid interface and contact angle analysis of organic liquid marbles formed from polymerized poly(ethylene glycol) diacrylate. a–e) SEM analysis of the solidified air‐liquid interface of liquid marbles formed using smooth particles (R_s_ = 0 nm) and supraparticles of surface roughness (R_s_) 120, 200, 310, and 485 nm, respectively. f) Contact angles of particles at the air‐liquid interface as a function of surface roughness, extracted from the SEM images. The contact angle value is an average of at least 5 different particle measurements for every sample. The error bar represents the standard deviation of these measurements.

To rationalize the relation between organic LM formation and surface roughness, we visualize the air‐liquid interface of an organic LM by interface fixing. In this case, we use poly(ethylene glycol) diacrylate as a liquid monomer (γ  =  41.8 mNm^−1^) mixed with a UV‐sensitive initiator (2‐hydroxy‐2‐methylpropiophenone, γ  =  38.3 mNm^−1^), form an LM, and induce polymerization by UV exposure. Within 5 min the liquid cures completely, forming solid polymer spheres (Figure S5, Supporting Information), with the stabilizing particles entrapped at the interface. **Figure** **4**a–e shows representative SEM images of the solidified interface, from which we extract the particle contact angles as a function of surface roughness. The results reveal a trend similar to that observed for the aqueous case, i.e., increasing the surface roughness leads to higher contact angles (Figure 4f). Consequently, for SP powders with similar surface chemistry, we conclude that the ability to form LMs from low‐surface‐tensions liquids is governed by their surface roughness, as it dictates the contact angle. Higher surface roughness translates to higher contact angles and thus reduced wetting of the powder, enabling LM formation from liquids with progressively lower surface tensions.

Next, we evaluate the mechanical stability of LMs upon impact by conducting drop tests. We place the formed LM on a plastic substrate, drop it from a defined height (Figure S6, Supporting Information) and record whether it remained intact or ruptured. The LM rupture can occur in two ways: localized attachment to the substrate via partial wetting or complete wetting of the substrate (Figure S7, Supporting Information). For our statistical analysis, we classify both partial and complete wetting as LM rupture. The test is conducted repeatedly (at least 10 times for each height) at height increments of 5 mm across the range from 5 to 60 mm, which enables us to correlate surface roughness with mechanical stability. We estimate the potential energy imparted to the LM during impact as:

(1) E=mgh

**Figure 5:**
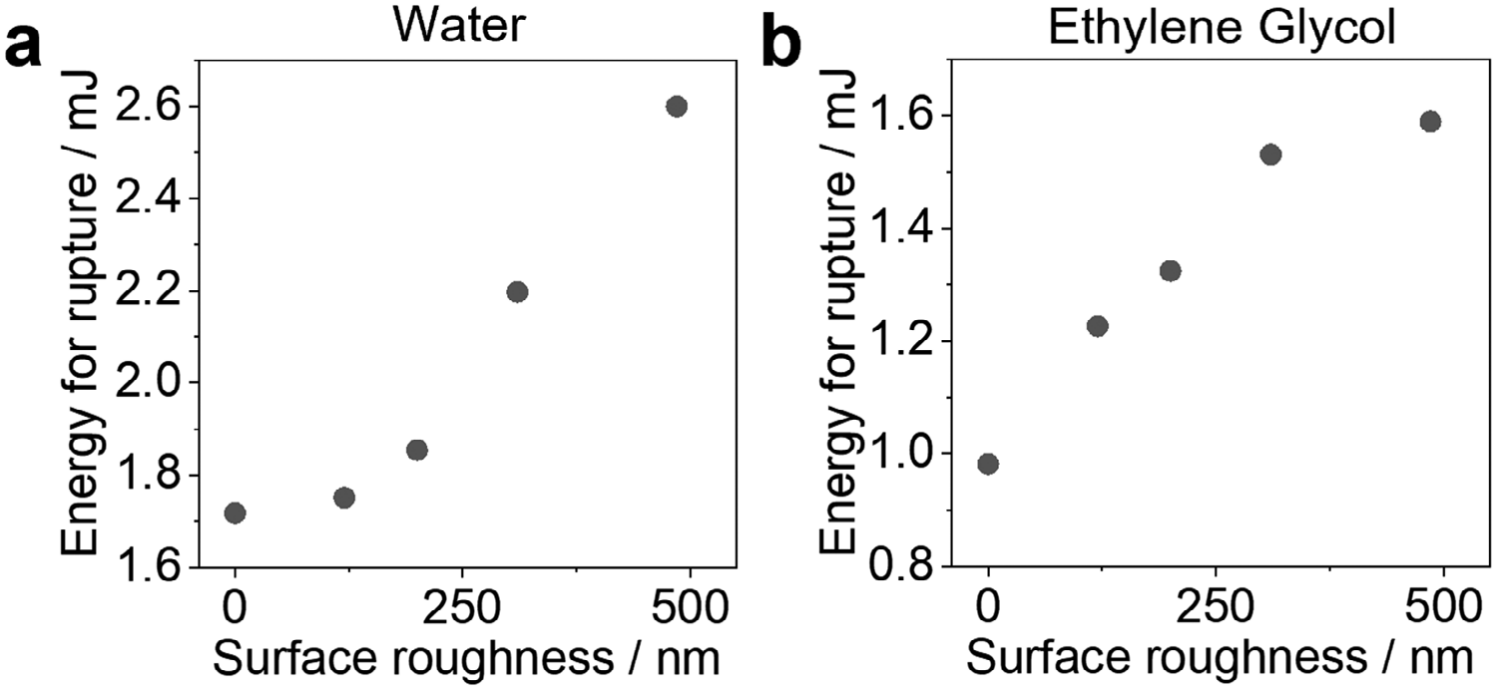
Quantification of mechanical stability of liquid marbles via drop testing. a) The energy required to rupture aqueous liquid marbles as a function of surface roughness of the stabilizing particles. b) Rupture energy of organic liquid marbles as a function of surface roughness, evaluated for ethylene glycol.

Here, *m* is the mass of LM, *g* is the gravitational acceleration, and the *h* is the height of the LM during the test. We define a rupture criterion as the height of the drop test at which at least 50% of the LMs rupture. From this criterion, we determine the energy required to rupture LMs for all particle systems (**Figure** **5**). Starting with aqueous LMs, we observe that the rupture energy increases with increasing particle surface roughness, with the 485 nm particles exhibiting the highest stability with a rupture energy of ≈2.6 mJ (Figure 5a). A similar trend is observed for organic LMs formed with ethylene glycol: larger particle surface roughness corresponds to higher rupture energies, i.e., improved mechanical stability (Figure 5b). The data also shows that organic LMs are less mechanically stable than their aqueous counterparts, as expected from the lower surface tension of the organic liquid, which decreases capillary attraction in between the particles. We attribute the increase in stability with larger surface roughness features to the higher contact angles observed for such rough particles (Figures 2f and 4f). Higher contact angles prevent particles from immersing deeper into the liquid at the interface, creating a larger gap between the inner liquid and the substrate that provides a cushion during mechanical impact. A similar effect has been previously reported for large, smooth particles, which show size‐dependent increases in mechanical stability.^[^ ^30^ ^]^ Additionally, we hypothesize that increased surface roughness promotes mechanical interlocking between particles adsorbed at the liquid interface, which reduces their interfacial mobility. This, in turn, enhances shell stiffness, resulting in LMs with greater impact resistance.

**Figure 6:**
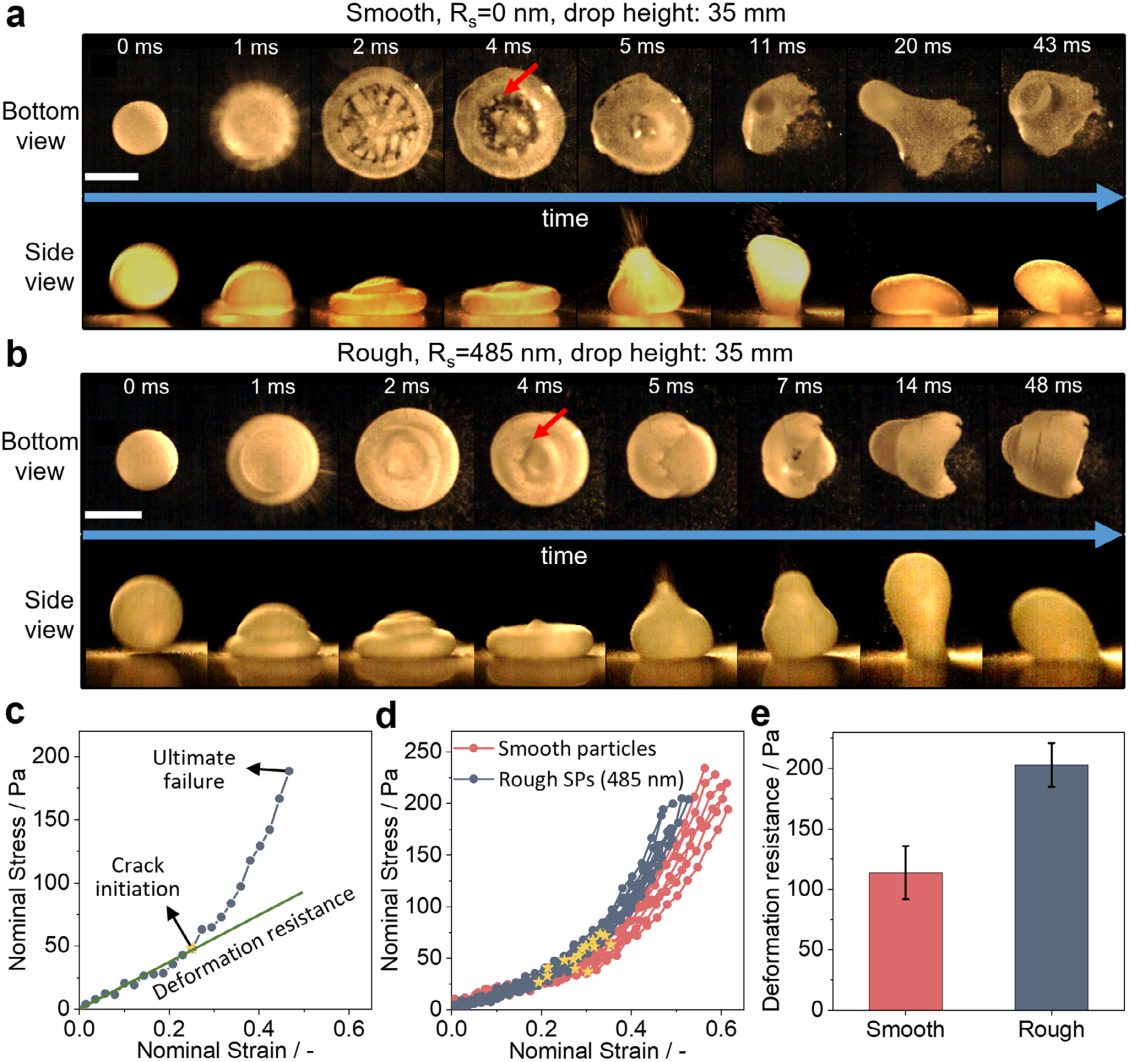
Characterization of mechanical properties of liquid marbles. a,b) High‐speed camera images showing deformation of liquid marbles during the drop test for the case of smooth (R_s_ = 0 nm) and rough (R_s_ = 485 nm) particles, respectively. Multiple cracks form in the interfacial particle layer for the smooth case. Arrows indicate the critical cracks that lead to rupture. c) Representative stress‐strain curve for liquid marbles obtained from controlled compression tests, exemplarily shown for a liquid marble stabilized by smooth particles. The deformation behavior changes after the formation of first cracks. d) Stress–strain curves from compression tests for liquid marble stabilized by smooth particles and rough supraparticles. Yellow stars indicate the point of crack initiation. Both types of particles show the same change in deformation behavior upon crack initiation. Smooth particles show a high strain before rupture. The compressive test was conducted for 7 LMs made from smooth particles and 10 LMs made from rough particles (R_s_ = 485 nm). e) Deformation resistance of liquid marbles formed by rough and smooth particles. Rough particles exhibit a higher deformation resistance indicating a stiffer shell. The deformation resistance values are obtained by taking an average of the slopes of the stress‐strain curves until the crack initiation point. The standard deviation of these is presented as the error bar. All scale bars are 2 mm.

To better understand the mechanical properties and, in particular, the role of surface roughness in stabilizing LMs, we directly compare the impact behavior of LMs formed by smooth particles and SPs with the largest surface roughness features (R_s_ = 485 nm). We image the deformation of LMs during the drop test using a high‐speed camera placed either at the side or below a bare glass substrate onto which the LM is dropped (details in Figure S8, Supporting Information). **Figure** **6**a,b shows the different stages of LM deformation during impact via time‐lapse images taken from Videos S1 and S2 (Supporting Information). Upon initial impact, the LM rapidly expands to a large extent and flattens, causing the opening of multiple cracks in the shell. Shortly after impact, a shock wave propagates through the LM, inducing oscillations. Some cracks grow beyond a critical threshold, resulting in localized wetting of the substrate. This wetting leads to LM failure by attachment and causes the LM to stretch and topple over.^[^ ^63^ ^]^ Meanwhile, the remaining portion of the LM recovers, and the shell reforms around the rest of the liquid. Qualitatively, this sequence of deformation is similar for smooth and rough particles. However, during the initial flattening on the substrate, LMs formed by smooth particles expand up to ≈100%, while LMs formed by rough particles only expanded up to ≈72%. This difference in expansion is accompanied by a difference in crack patterns: the shell formed by smooth particles exhibits many large cracks, while the shell formed by rough particles remains largely intact. This different resistance to expansion indicates stronger cohesion between the interfacially‐adsorbed rough particles and a stiffer shell. The direct observation thus corroborates the drop test data which shows that the rough particles require higher energy to rupture (Figure 5a).

We also observe cases where the LM undergoes complete wetting of the substrate or complete recovery, depending on the dropping height. At larger heights, the impact energy is so high that the LM's outer rim expands excessively, exposing a particle‐free liquid surface that wets the substrate entirely (Figure S9 and Videos S3 and S4, Supporting Information). In contrast, at lower heights, the impact energy is sufficiently reduced that the interfacial particle layer can re‐form after the initial expansion, enabling the LM to recover (Figure S10 and Videos S5 and S6, Supporting Information). Notably, LMs formed from smooth particles exhibit plastic deformation, resulting in an oblate‐like shape after impact. This behavior may be attributed to the greater mobility of smooth particles and contact sliding.^[^ ^64^, ^65^ ^]^ Conversely, LMs formed from rough particles retain their spherical shape, even after impact, likely due to limited particle mobility at the interface.

We perform compression tests to support the observations of the drop tests and evaluate the mechanical properties under more controlled conditions. For these tests, the LM is compressed between two glass plates while being observed with a camera from both the top and side, as shown schematically in Figure S11 (Supporting Information). We use a fine weighing balance to record force values during compression,^[^ ^32^ ^]^ which is applied at a constant speed of 15 µm s^−1^. Videos S7 and S8 (Supporting Information) show the compression process for smooth and rough LMs, respectively, where we observe that the LMs rupture near the edges under compression.^[^ ^66^ ^]^ Notably, in these videos, a clear motion of individual particles and rearrangement processes are observed for LMs formed by smooth particles, but not for rough particles. This observation corroborates the mechanical interlocking and thus rationalizes the increased impact resistance. During compression, the shape of the LMs changes continuously, leading to a dynamically changing contact area which is difficult to measure reliably. We therefore calculate nominal strain by dividing the displacement with the initial diameter of the LM and nominal stress by dividing force with the cross‐sectional area of the uncompressed LM. These calculations yield the stress‐strain curve exemplarily shown for an LM stabilized by smooth particles in Figure 6c. The curve exhibits two distinct regimes.^[^ ^67^ ^]^ Initially, the stress increases linearly with strain until the first cracks appear, marked as a star in the stress‐strain curves. Note that this point of crack initiation is determined visually by observing the LM during compression, leading to a potential overestimation as cracks opening up on the backside of the LM would not be detected. The mechanics in this linear regime are predominantly influenced by the surface tension of the liquid and the layer of adsorbed particles.^[^ ^32^, ^67^, ^68^ ^]^ After crack initiation, further compression causes these cracks to grow, resulting in an exponential increase in stress with strain. We attribute this change in behavior to the higher energy required to create new, bare surface areas of water as the cracks expand. The formation of these new surfaces increases the effective surface tension of the LM, necessitating greater stress for further deformation.^[^ ^69^ ^]^

We compare the compressive deformation of LMs formed with smooth and rough particles and observe that both show a similar two‐stage deformation behavior (Figure 6d) and a comparable stress at rupture. LMs formed by smooth particles, however, display a higher strain at rupture indicating greater particle mobility at the interface and the potential for rearrangement under slow compression. This hypothesis is further supported by our observation of relative movement among smooth particles on LM surface during the initial compression phase, before any visible cracks form (Video S7, Supporting Information). This increased mobility also contributes to the formation of numerous cracks in the smooth particle shell, observed during the drop tests in Figure 6a. To quantify these effects, we evaluate the slope of the linear region in the stress‐strain curve, which is commonly used to obtain Young's moduli for elastic materials. For LMs, we interpret this slope as deformation resistance, representing the ability of the LM to resist deformation via the interfacially‐adsorbed particle layer before crack initiation (Figure 6c). The deformation resistance can thus be considered a measure of the stiffness of the particle shell. Notably, LMs stabilized by rough particles exhibit a deformation resistance of 203 ± 18.2 Pa, compared to 114 ± 22.1 Pa for LMs formed by smooth particles (Figure 6e). This increased deformation resistance further evidences the mechanical interlocking of the rough particles, which leads to an enhanced stiffness of the interfacial particle layer. This interlocking reduces particle mobility, as observed in the videos upon compression, resulting in a lower strain at fracture during slow compression, but enhances impact resistance during drop testing by increasing resistance to deformation and crack initiation.

## 3 Conclusion

We systematically investigate the impact of particle surface roughness on LM formation and mechanical stability using SPs with controlled surface roughness features. Larger surface roughness increases the particle contact angle at the air‐liquid interface. We demonstrate that this increase is consistent across aqueous and organic liquids, enabling the formation of LMs with progressively lower surface‐tension liquids, with thresholds decreasing from ≈40 mN m^−1^ for smooth particles to ≈22.9 mN m^−1^ for rough particles. Rough particles enhance the mechanical stability of LMs by mechanical interlocking that reduces interfacial mobility, as demonstrated by higher rupture energies and reduced expansion under impact. Moreover, the decreased mobility allows LMs from rough particles to retain their spherical shape after impact, contrasting with the plastic deformation and oblate shapes observed for LMs stabilized by smooth particles. Compression tests reveal a two‐stage deformation process: an initial linear region that transitions into exponential stress increase after crack initiation. As a result of interlocking, LMs from rough particles exhibit an increased deformation resistance and a lower strain at rupture compared to LMs from smooth particles. Our study emphasizes the critical role of particle surface roughness in determining the formation and mechanical stability of LMs and provides insights for designing robust LMs tailored for advanced applications such as sensors, microfluidics and soft materials.

## 4 Experimental Section

### Materials

Absolute ethanol (≥99.8%, NORMAPUR, VWR), ammonium hydroxide (NH_4_OH, 25%, NORMAPUR, VWR), tetraethyl‐orthosilicate (TEOS, ≥99%, GPR RECTAPUR, VWR), technical ethanol (96%, VWR), 1H,1H,2H,2H‐heptadecafluorodecyl‐trichlorosilane (≥97%, FUJIFILM Wako), *n*‐hexane (≥98%, Sigma–Aldrich), ethylene glycol (≥99%, Sigma–Aldrich), benzyl benzoate (≥99%, FUJIFILM Wako), *n*‐pentadecane (≥97%, FUJIFILM Wako), *n*‐tridecane (≥99%, FUJIFILM Wako), *n*‐dodecane (≥99%, Sigma–Aldrich), *n*‐decane (≥99%, FUJIFILM Wako), *n*‐nonane (≥98%, FUJIFILM Wako), SuperGlue (Toagosei Co. Ltd), poly(ethylene glycol) diacrylate (Mn 700, Sigma–Aldrich), 2‐hydroxy‐2‐methylpropiophenone (97%, Sigma–Aldrich), smooth silica particles (2, 6, 11, 20 µm, HIPRESICA N2N) were all used as received. De‐ionized water was used for all the experiments, purified using Purelab Flex 2 (Elga Veolia, 18.2 MΩ cm).

### Synthesis of Supraparticles (SPs)

SPs were prepared as previously described in literature.^[^ ^49^ ^]^ First, colloidal silica primary particles were synthesized using the Stöber method according to a modified protocol from literature.^[^ ^70^ ^]^ In a typical procedure, water, absolute ethanol and NH_4_OH were mixed in a two liter, three‐neck round bottom flask, stirred at 300 rpm. Afterward, TEOS was added under constant stirring, and the solution was stirred for 16 h at room temperature. The reaction volume was 1.5 L and the concentration of the ethanolic synthesis solution was 8 m water, 0.15–2 m NH_3,_ and 0.3 m TEOS. The transparent solution turned white overnight. The synthesized particles were separated from the solution via centrifugation, followed by cleaning with a mixture of water‐ethanol (50:50 volume) 3 times and only water 2 times. At the end, they were dispersed in water at a solid concentration of 45 wt.%. The primary particle size was adjusted by varying the concentration of NH_3_: 0.21 m for 240 nm, 0.45 m for 400 nm, 1.5 m for 620 nm and 2.5 m for 970 nm.

Subsequently, the SPs were fabricated by spray‐drying the silica colloids using a BÜCHI spray B290‐Mini spray‐dryer, under nitrogen atmosphere. A co‐current flow two‐fluid nozzle (Ø = 1.4 mm) was used to atomize the colloid feed at a gas flow of 357 L h^−1^ and feed flow of 3.4 mL min^−1^. The aspirator flow was set to 35 m^3^ h^−1^ with an inlet temperature of 130 °C. The spray‐dried SP powders were sintered at 1000 °C (2 °C min^−1^) for 15 h to improve their mechanical stability.

Smooth particles, obtained commercially, were mixed in a weight ratio of 1 (2 µm): 6.6 (6 µm): 35 (11 µm): 58.4 (20 µm) to replicate a size distribution similar to that of SP powders. This specific composition was based on the particle size distribution of the SPs (shown in Figure S2, Supporting Information). From the volume distribution, the approximate volume ratio of the available sizes of commercial smooth particles (2, 6, 11, and 20 µm) was extracted and used as the composition.

### Characterization of Particle Size

The size distributions of silica primary particles were measured via dynamic light scattering (DLS) using ZetaSizer Nano (Malvern Panalytical). Size distributions of SPs were measured via laser diffraction using MasterSizer 3000/Hydro 3000S (Malvern Panalytical).

### Surface Modification of Particles

All powder samples were rendered non‐wetting by treating with a silane coupling agent (heptadecafluorodecyl‐trichlorosilane). In a typical procedure, the silane was added to *n*‐hexane (0.5 vol%), followed by the addition of powder (0.02 g mL^−1^) and left on an orbital shaker for 4 h. Afterward, the modified particles were rinsed three times with *n*‐hexane. The particles were then dried in atmosphere for 24 h at room temperature and left under vacuum overnight.

### Fabrication of Liquid Marbles (LMs)

The LMs were formed by placing the sample of powder onto a plastic petri‐dish making a powder bed. Next, 5 µL of liquid was partially ejected so that it rested at the tip of the pipette, and then dipped into the powder bed before being fully dispensed. This step ensures that the droplet is sufficiently coated, preventing it from sticking to the bowl when released. The droplet was then rolled in the powder bed for 40 s to completely coat its surface with particles, followed by an additional 30 s of rolling on a bare glass surface to remove any excess particles. The prepared LM was carefully picked up using a spatula, ensuring that the spatula's edge did not damage the LM, and then used in further experiments.

### Interface Fixing and Visualization

The interface of aqueous LMs was fixed as previously described in literature.^[^ ^56^ ^]^ For this, Super‐Glue was vaporized under a glass container placed on a heating plate at 50 °C. The LM was then also placed under this glass container exposing it to Super‐Glue vapor to initiate anionic polymerization at the interface. This is schematically shown in Figure S3 (Supporting Information). Before analysis, the water inside the LM was allowed to evaporate.

For organic LMs, poly(ethylene glycol) diacrylate monomer and a UV sensitive initiator, 2‐hydroxy‐2‐methylpropiophenone was used, mixed in a weight ratio of 10:1. Immediately after mixing, the liquid is used to form LM which is subsequently exposed to UV light for 5 min to fully cure the polymer (Figure S5, Supporting Information).

Once solidified, the interfaces were imaged in SEM (Gemini 500, Zeiss) with an SE2 detector, at an acceleration voltage of 1 kV and aperture size of 15 µm. The working distance was kept at 6 mm. The particle boundary and the interface are manually defined as shown in Figure S4 (Supporting Information) by a sphere and a line respectively. A tangent is drawn at the air‐liquid‐particle contact point, also shown in Figure S4 (Supporting Information). The angle formed between this tangent and the polymerized liquid interface is then determined using the “angle tool” in the image analysis software Image J.

### Drop Testing of LMs

The prepared LM sample was placed at the center of a rigid, flat plastic substrate. Keeping the substrate horizontal, it was dropped from heights ranging between 5  and 60 mm (Figure S6, Supporting Information). After each test, the LM's structural integrity was assessed by looking for signs of wetting. If the LM remained stationary and exhibited no signs of movement, it was considered ruptured.

To observe the LM during impact, they were dropped on a bare glass substrate. A high‐speed camera (Motion BLITZ) was placed either under the glass slide or to the side of it to image the LM upon impact. The LMs were dropped using a hydrophobic substrate as shown schematically in Figure S8 (Supporting Information). The high‐speed camera was set to a frame rate of 1865 fps. The degree of expansion of the LM was determined by calculating the difference between its diameter before testing and its maximum diameter during impact, then dividing this difference by the initial diameter.

### Compressive Testing of LMs

The LM was tested under compression by placing it on a glass slide which was placed on top of a precision scale (Sartorius AX224) and a glass slide attached to a syringe pump (Sigma 1000, Cronus) via a rigid metal rod was used for compression (Figure S11, Supporting Information). The position of the LM, and the syringe pump was adjusted so that the glass slide would lower onto the LM. The syringe pump was set to a constant velocity of 15 µm s^−1^. Cameras (Alpha 6400, Sony) equipped with a macro lens were used, positioned on top and side of the LM, to observe the compression process.

### Statistical Analysis

The contact angle was measured for at least 5 different particles for every sample. These measurements were then averaged and presented in plots in Figures 2 and 4 together with the standard deviation as the error bar. At least 10 drop tests were conducted at each height for every powder sample and both the liquids (water, and ethylene glycol) to quantify the impact stability of LMs. The compressive testing of LMs was conducted for 7 LMs made from smooth particles and 10 LMs made from rough SPs (R_s_ = 485 nm). The deformation resistance values presented in Figure 6e were obtained by taking an average of the slopes of the stress‐strain curves until the crack initiation point. The standard deviation of these is presented as the error bar. The particle size distributions shown in Figure S2 (Supporting Information) are normalized by dividing with the highest value.

## Conflict of Interest

The authors declare no conflict of interest.

## Author Contributions

U.S. designed and realized the experiments, synthesized all the materials, performed characterization, mechanical testing (drop tests and compression tests), analysis, interpretation, wrote the original draft, reviewed, and edited the final manuscript. C.K. and K.K. performed drop tests and compression tests and reviewed the final manuscript. N.V. and S.F. acquired the funding, reviewed, and edited the final manuscript. U.S., S.F., and N.V. conceptualized and supervised all the research work. All authors contributed to the discussion of the results presented.

## Supporting information



Supporting Information

Supplemental Video 1

Supplemental Video 2

Supplemental Video 3

Supplemental Video 4

Supplemental Video 5

Supplemental Video 6

Supplemental Video 7

Supplemental Video 8

## Data Availability

The data that support the findings of this study are openly available in [zenodo] at [https://doi.org/10.5281/zenodo.14961797], reference number [14961797].
