# Supplementary material for: Rough and Tough: How Particle Surface Roughness Affects Liquid Marble Formation and Stability
Source: Adv Sci (Weinh). 2025 Apr 7;12(25):2501378. doi: 10.1002/advs.202501378 (PMC12224969; doi:10.1002/advs.202501378)
Supplement: Supplementary file 1 — Supporting Information [file ADVS-12-2501378-s008.pdf]

ADVANCED SCIENCE
Open Access

Supporting Information

for *Adv. Sci.*, DOI 10.1002/advs.202501378

Rough and Tough: How Particle Surface Roughness Affects Liquid Marble Formation and Stability

Umair Sultan, Celin Kotulla, Kall Kefle, Syuji Fujii and Nicolas Vogel**

Supporting Information

Rough and Tough: How particle surface roughness affects liquid marble formation and stability

Umair Sultan, Celin Kotulla, Kall Kefle, Syuji Fujii, Nicolas Vogel**

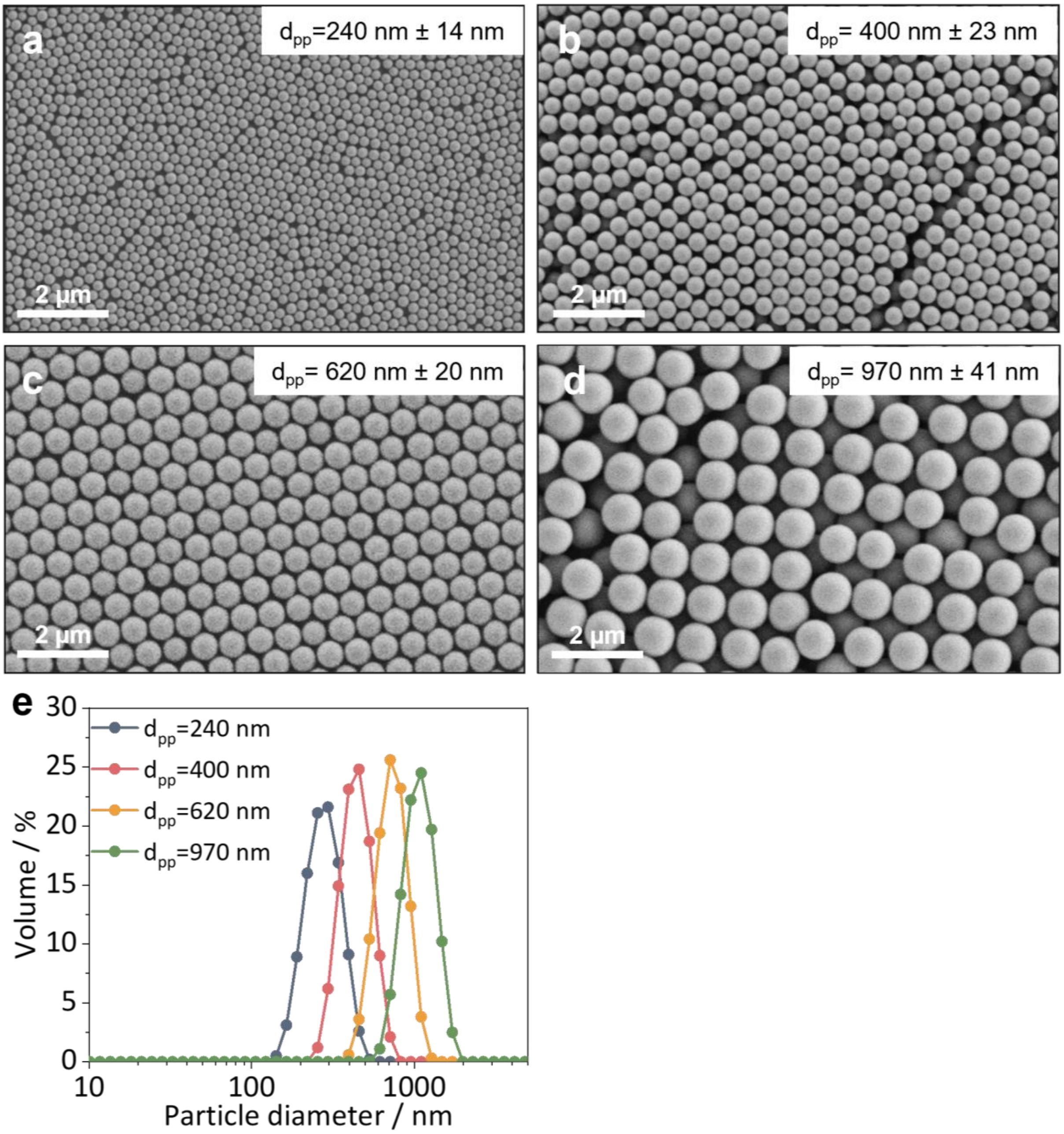

Figure S1. Colloidal silica primary particles. (a-d) SEM images of silica primary particles with average particle diameter of (a) 240 nm, (b) 400 nm, (c) 620 nm, and (d) 970 nm. (e) The corresponding particle size distributions of the synthesized silica primary particles.

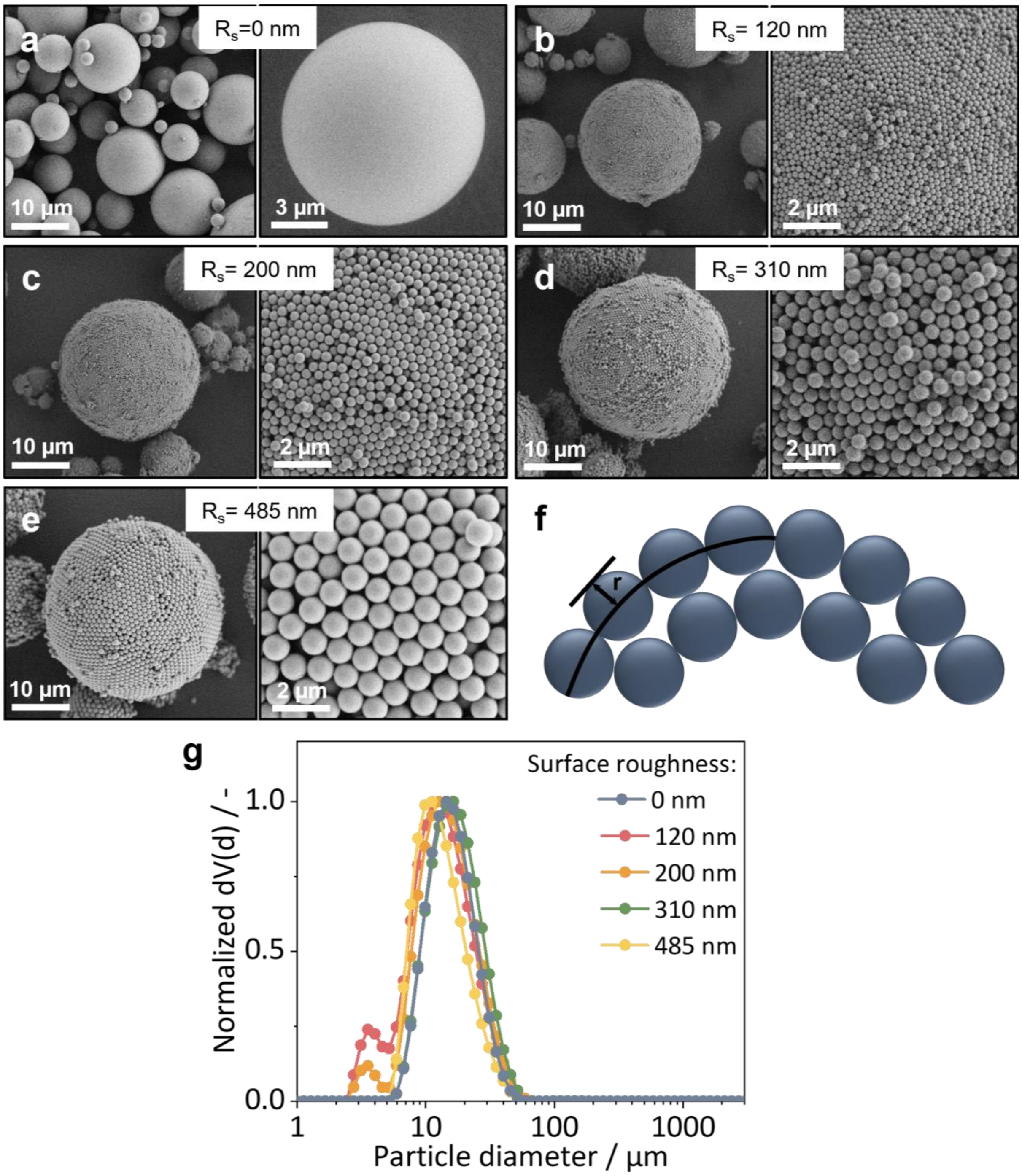

Figure S2. Characterization of the stabilizer silica particles used for making liquid marbles. (a-e) Low and high magnification SEM images of (a) smooth particles with 0 nm surface roughness (mixture of 2 µm, 6 µm, 11 µm, and 20 µm commercial particles mixed in a weight ratio of 1:6.6:35:58.4, respectively), and supraparticles (SPs) with surface roughness (R_s) of (b) 120 nm, (c) 200 nm, (d) 310 nm, (e) 485 nm. A few satellite primary particles can be observed on the surface of the SPs, a common feature of SPs fabricated via spray drying. This occurs due to the dynamic nature of the spray drying process, where, during drying, SPs collide with the chamber walls. These collisions generate free primary particles, which subsequently adhere to the surface of the formed SPs. (f) Schematic illustration of the supraparticle surface showing that radius r of the primary particles defines the surface roughness. (g) The particle size distributions of the smooth and rough particles. The particle size distributions are normalized by dividing with the largest value. The composition used for the commercial smooth particles is based on the volume distribution of SPs shown here.

Table S1. Gravimetric analysis of liquid marbles. Number of hexagonally closed packed particles on the surface of a spherical liquid marble is given by $N = \left(\frac{4\pi {R_{LM}}^2}{\pi {R_p}^2}\right) 0.91$, where, R_{LM} is the radius of the liquid marble and R_p is the radius of the adsorbed particles.

**Particle type (surface roughness)**	**Mass of one LM (mg)**	**Particle loading on LM surface (wt %)**	**No. of adsorbed particles in a monolayer[a]**	**No. of actual adsorbed particles[b]**	**No. of layers of adsorbed particles[c]**
0 nm, smooth	5.05	5.94	165,786	128,044	1.29
120 nm	5.02	5.18	143,681	129,096	1.11
200 nm	5.03	6.26	174,075	128,785	1.35
310 nm	4.91	5.60	151,970	121,335	1.25
485 nm	4.97	6.23	171,312	128,214	1.33

[a] Calculated using the equation above, assuming spherical shape of liquid marbles and hexagonal close packing of particles at the air-water interface. [b] Determined gravimetrically. [c] Calculated by dividing no. of adsorbed particles in a monlayer with no. of actual adsorbed particles.

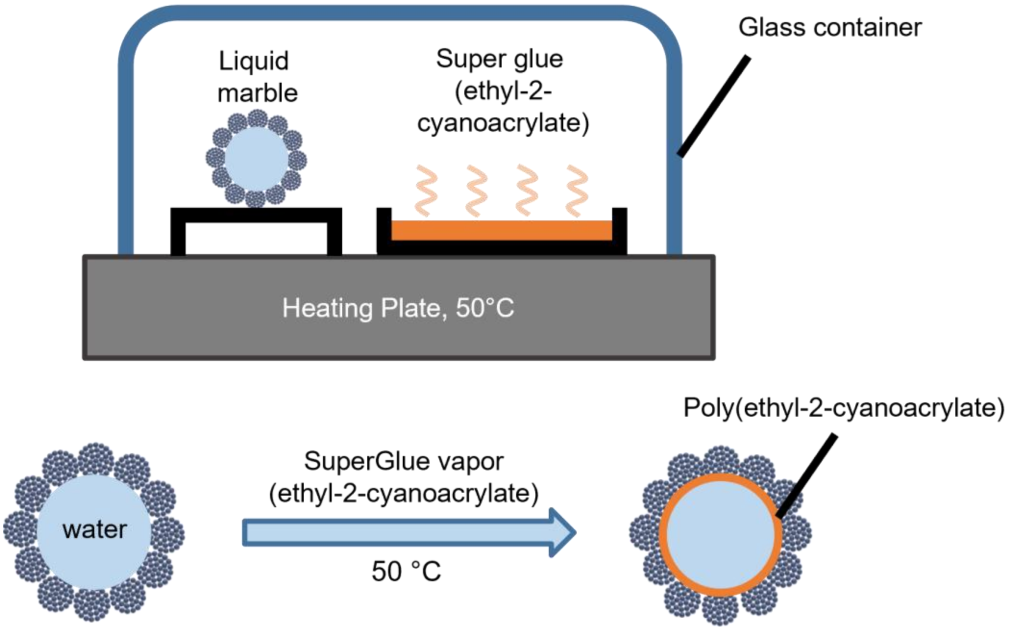

Figure S3. Schematic illustration of the interface fixing process for aqueous liquid marbles. The aqueous liquid marble is enclosed under a glass container together with Super-Glue (ethyl-2-cyanoacrylate) vapor, causing anionic polymerization at the liquid marble interface that fixes the position of the particles.

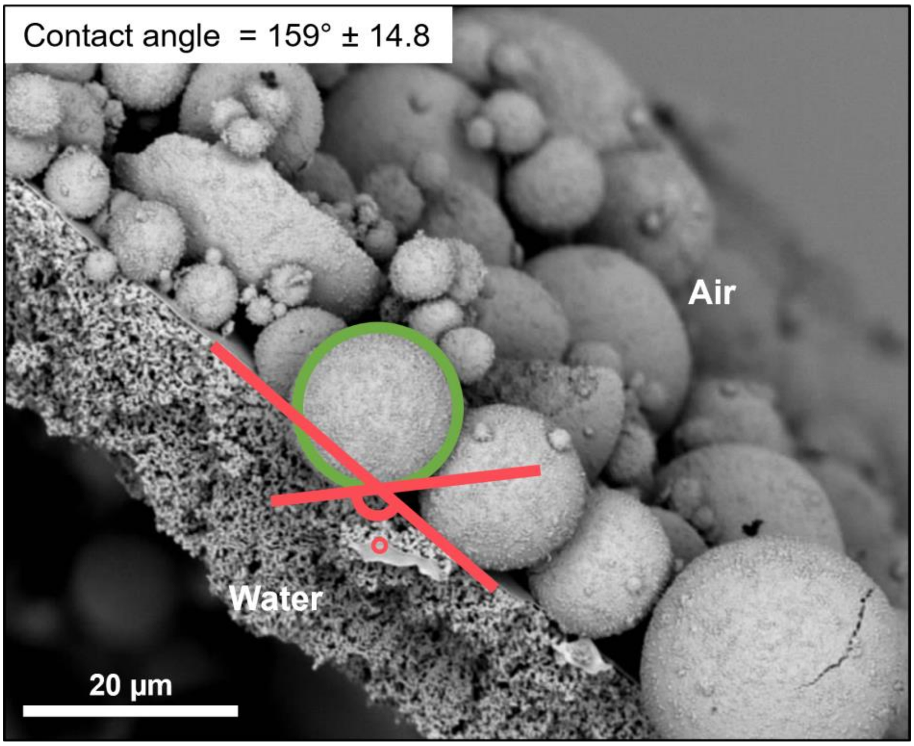

Figure S4. Illustration of the geometric estimation of contact angle of particles from SEM images of liquid marble interface. Exemplarily shown for supraparticle with surface roughness (R_S) of 200 nm.

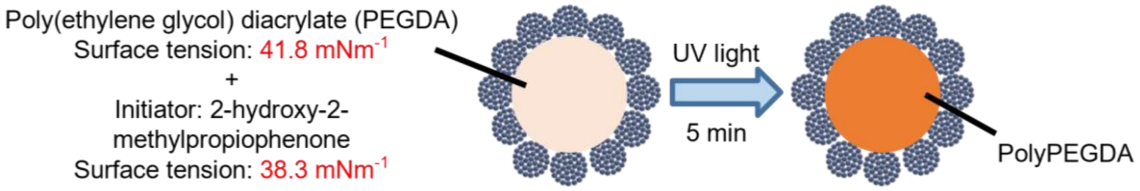

Figure S5. Interface fixing of organic liquid marbles. Organic liquid marbles formed from a mixture of poly(ethyleneglycol) diacrylate and UV sensitive initiator, 2-hydroxy-2-methylpropiophenone are cured under UV light to fix the position of particles at the interface of the liquid marble.

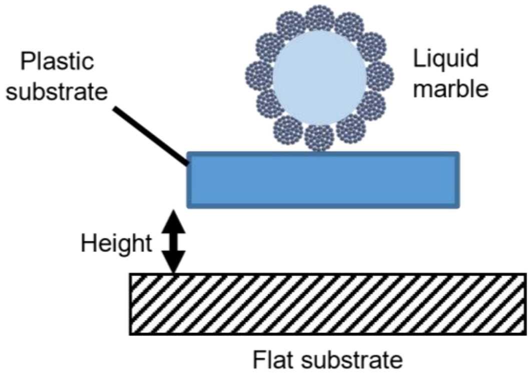

Figure S6. Schematic illustration of the liquid marble drop test. The liquid marble is placed on a plastic substrate and dropped from a certain height while keeping the substrate horizontal.

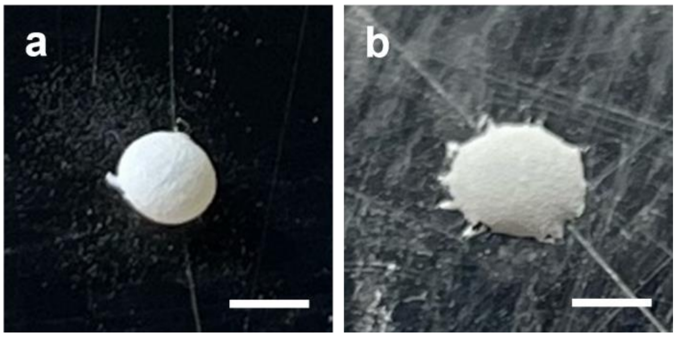

Figure S7. Liquid marble rupture upon impact during drop test. (a, b) Photographs of dropped liquid marbles showing (a) rupture by partial wetting and (b) rupture by complete wetting of the substrate. All scale bars are 2 mm.

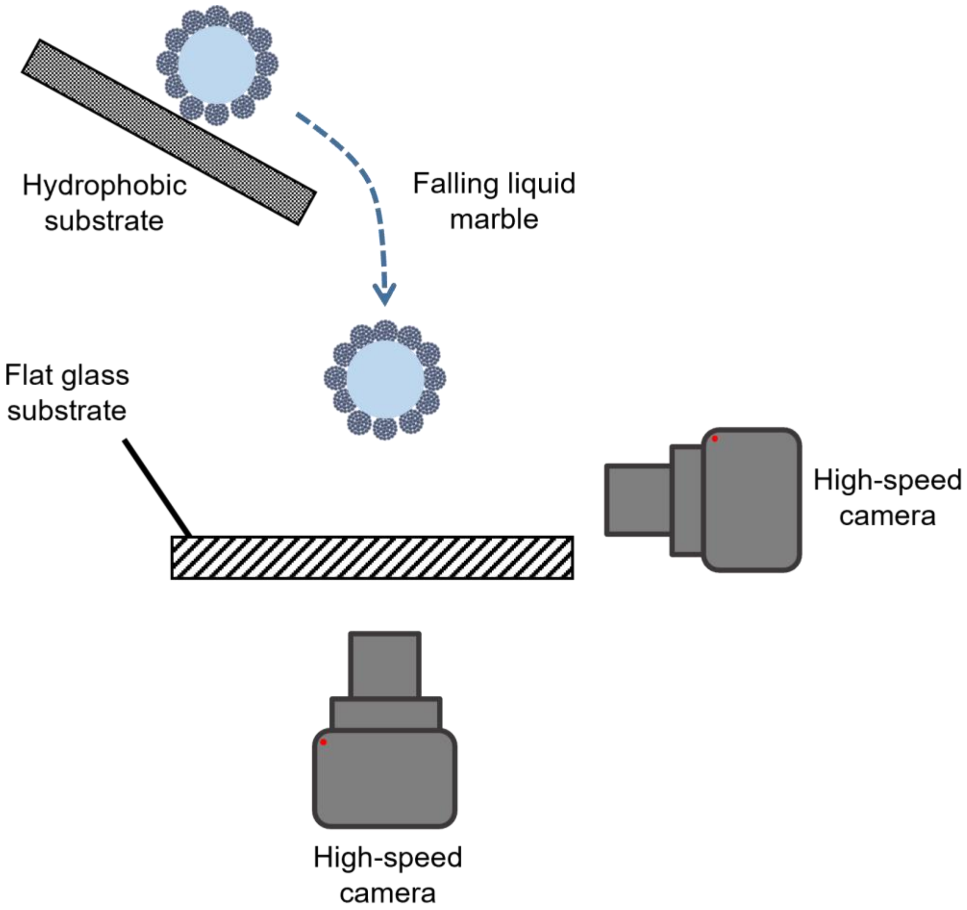

Figure S8. Schematic illustration of the observation of liquid marble during drop test. Liquid marble is dropped onto a glass substrate using a hydrophobic surface. A high-speed camera, placed either below the glass or at the side of it, is used to image the liquid marble upon impact.

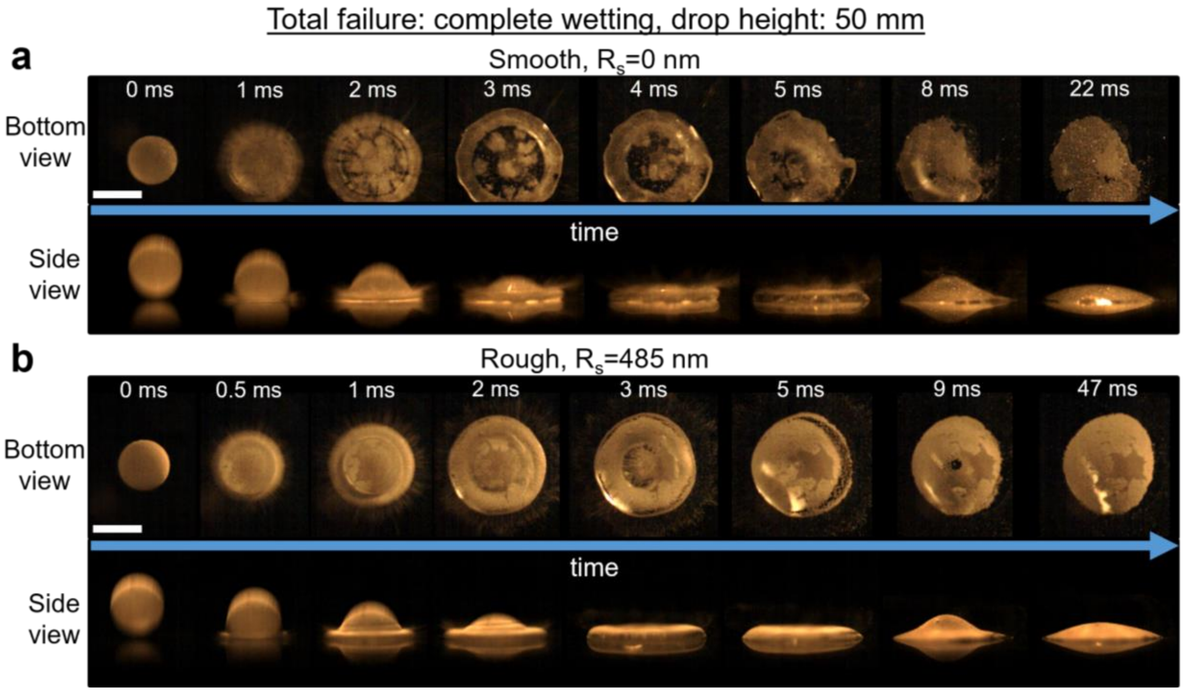

Figure S9. Observation of liquid marble failure by complete wetting during drop test. High-speed camera images of failure during drop test for liquid marble made from: (a) smooth and (b) rough particles. The larger drop height results in a greater expansion of the liquid marble, forming a large crack around the rim, leading to water wetting the substrate completely. All scale bars are 2 mm.

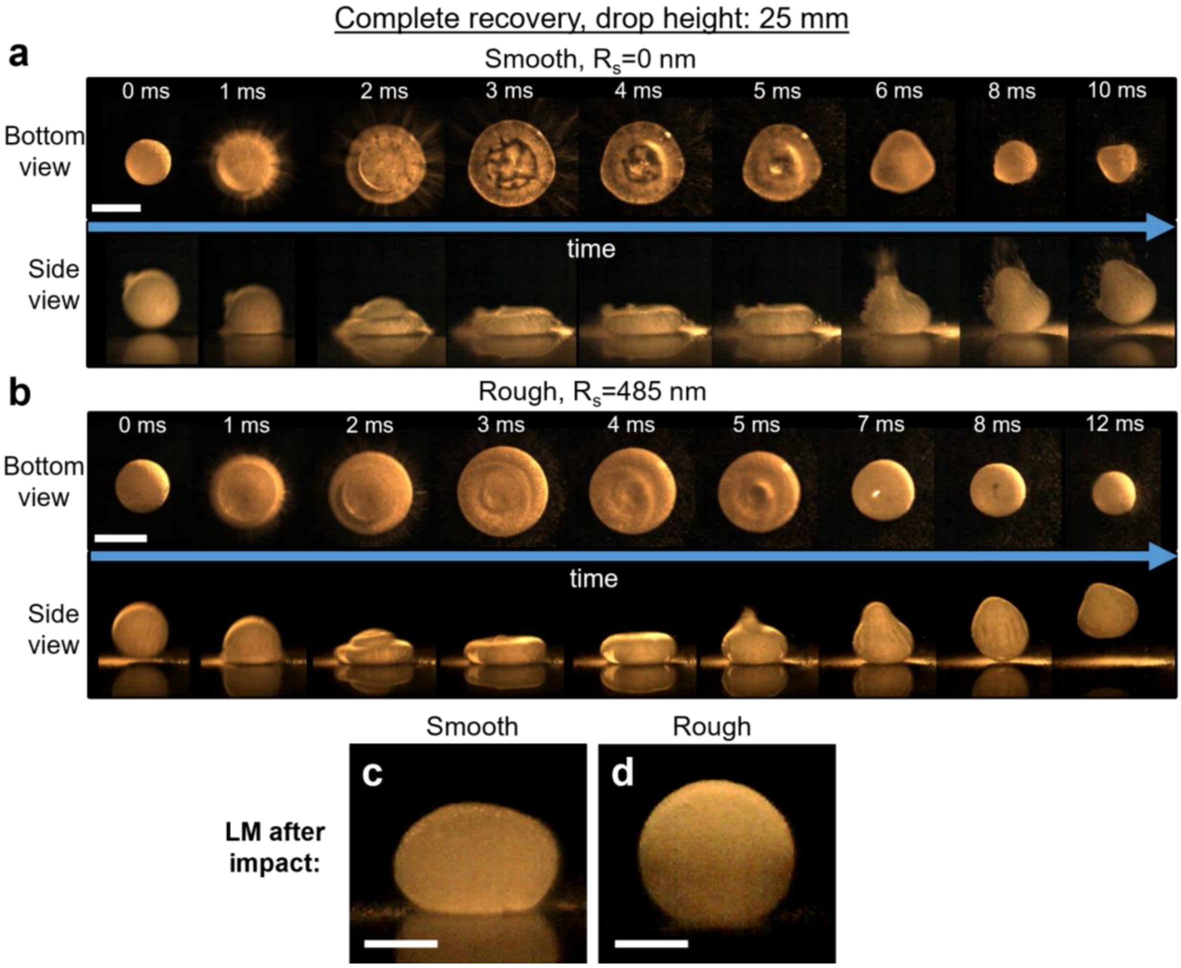

Figure S10. Observation of liquid marble recovery during drop test. High-speed camera images of liquid marble recovery after impact for the case of: (a) smooth and (b) rough particles. The scale bars 2 mm. The liquid marbles recover completely after impact due to the smaller drop height. Liquid marble made from smooth particles (c) however shows plastic deformation, resulting in an oblate-like shape after impact. While the liquid marble from rough particles (d) maintains its spherical shape. The scale bars in (c) and (d) are 1 mm.

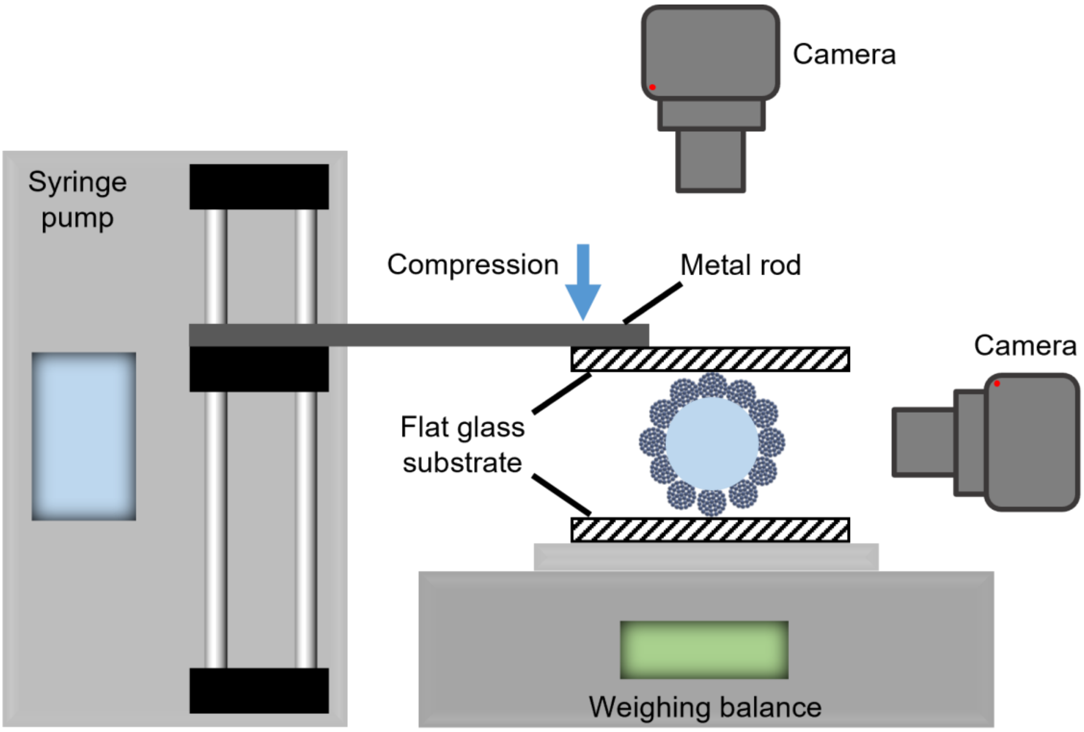

Figure S11. Schematic illustration of the liquid marble compression test. The liquid marble is placed on a glass substrate on top of a weighing balance. A glass substrate attached to a syringe pump via a metal rod is used to compress the liquid marble at a constant velocity. During compression the liquid marble is observed from the top and the side using cameras.

Supplementary Videos Captions

Video S1. High-speed camera video of smooth-particle (R_s=0 nm) LM dropped from a height of 35 mm.

Video S2. High-speed camera video of rough-particle (R_s=485 nm) LM dropped from a height of 35 mm.

Video S3. High-speed camera video of smooth-particle (R_s=0 nm) LM dropped from a height of 50 mm.

Video S4. High-speed camera video of rough-particle (R_s=485 nm) LM dropped from a height of 50 mm.

Video S5. High-speed camera video of smooth-particle (R_s=0 nm) LM dropped from a height of 25 mm.

Video S6. High-speed camera video of rough-particle (R_s=485 nm) LM dropped from a height of 25 mm.

Video S7. Video of a smooth-particle (R_s=0 nm) LM undergoing compression.

Video S8. Video of a rough-particle (R_s=485 nm) LM undergoing compression.
